# How do complex animal signals evolve?

**DOI:** 10.1371/journal.pbio.3000093

**Published:** 2018-12-17

**Authors:** Chad M. Eliason

**Affiliations:** Integrative Research Center, Field Museum of Natural History, Chicago, Illinois, United States of America

## Abstract

Animal signals—involving combinations of acoustic, chemical, visual, and behavioral cues—are among the most diverse traits in nature. Testing hypotheses about signal evolution has been hampered by difficulties with comparing highly divergent signals among species. In this Primer, I describe recent advances in capturing signals and studying their evolution. I highlight new findings using an information theory–based approach to quantifying signal variation in the diverse birds-of-paradise. Growing access to signal databases in tandem with development of new analytical tools will open up new avenues for studying the proximate mechanisms and ultimate evolutionary causes of signal variation, both within and among species.

## Abstract 2

Animals communicate with each other in diverse ways–using chemicals, sounds, colors and movement. This Primer explores research on animal signals and highlights the need for advances in studying how complex signals evolve, such as that described in a recent PLOS Biology paper.

## History of the study of animal signals

Animals communicate with each other in diverse ways, including chemicals, sounds, colors, and movement. Development of a theoretical framework for how signal traits coevolve with preferences in the 1990s led to a burst of research on the mechanisms and diversity of animal signals [1]. Today, animal signals provide a rich arena for integrative biological research, encompassing physiologists interested in the physical or chemical mechanisms producing signals [2], developmental biologists interested in the genetic bases for signals [3], and neurobiologists studying the processes involved in receiving signals [4], as well as behavioral ecologists and evolutionary biologists studying the causes and consequences of signal divergence among species [5].

## Drivers of signal diversity and complexity

Elaborate ornaments and signals—like peacock trains and anole dewlaps—are among the most diverse traits in nature. Signal traits provide unique opportunities for studying trait evolution more generally for two important reasons. First, animal signals are complex, varying spatially (for example, color patterns in butterfly wings), temporally (for example, notes in a bird's song), and qualitatively (for example, combined behavioral and visual components of the mating dances of peacock spiders). Second, signal traits are a classic system for studying how sexual selection works because of the increased strength and constancy of sexual selection compared to natural selection [6] and the greater potential for rapid trait divergence if traits and preferences are genetically linked [7]. The idea that sexual selection drives signal diversity, as first emphasized by West-Eberhard [7], has been an important yet controversial idea in biology. For example, Seddon and colleagues [8] showed that sexual selection promotes trait divergence during speciation, while recent theoretical work [9] suggests that sexual selection might instead limit signal divergence in some contexts. This disagreement might arise, in part, from our lack of understanding of how signals are produced or whether and how signals are evolving under natural and/or sexual selection. A third explanation for disagreement over the role of social selection in driving signal diversity might be our lack of understanding of the interrelationships between different aspects of complex signals [10–12].

## Advances in measuring signal diversity

Early research into animal signals involved the use of standard color swatches or phonetic renderings of bird songs (for example, the "whip-poor-will" call of the eastern whip-poor-will). In the last 30 years, technological advances in, and falling prices of, devices to quantify signals numerically (for example, portable spectrophotometers, high-speed digital cameras, or self-powered magnetic recorders) [13] have driven an explosion of research into signal diversity in nature [1]. Now is an exciting time to be an evolutionary biologist, with vast amounts of life-history data collected through painstaking fieldwork [14], as well as acoustic (for example, xeno-canto.org, macaulaylibrary.org), colorimetric (for example, projectplumage.org), and chemical signal data [15] becoming increasingly available through online data repositories. We are at the precipice of breakthroughs in the integration of these different data streams to answer new questions about the evolution of complex signals. However, despite recent advances in capturing signals in nature and making them readily available, researchers face new challenges with comparing highly divergent signals among species and analyzing them in an evolutionary framework.

## Evolution of signal complexity in birds-of-paradise

**Fig 1:**
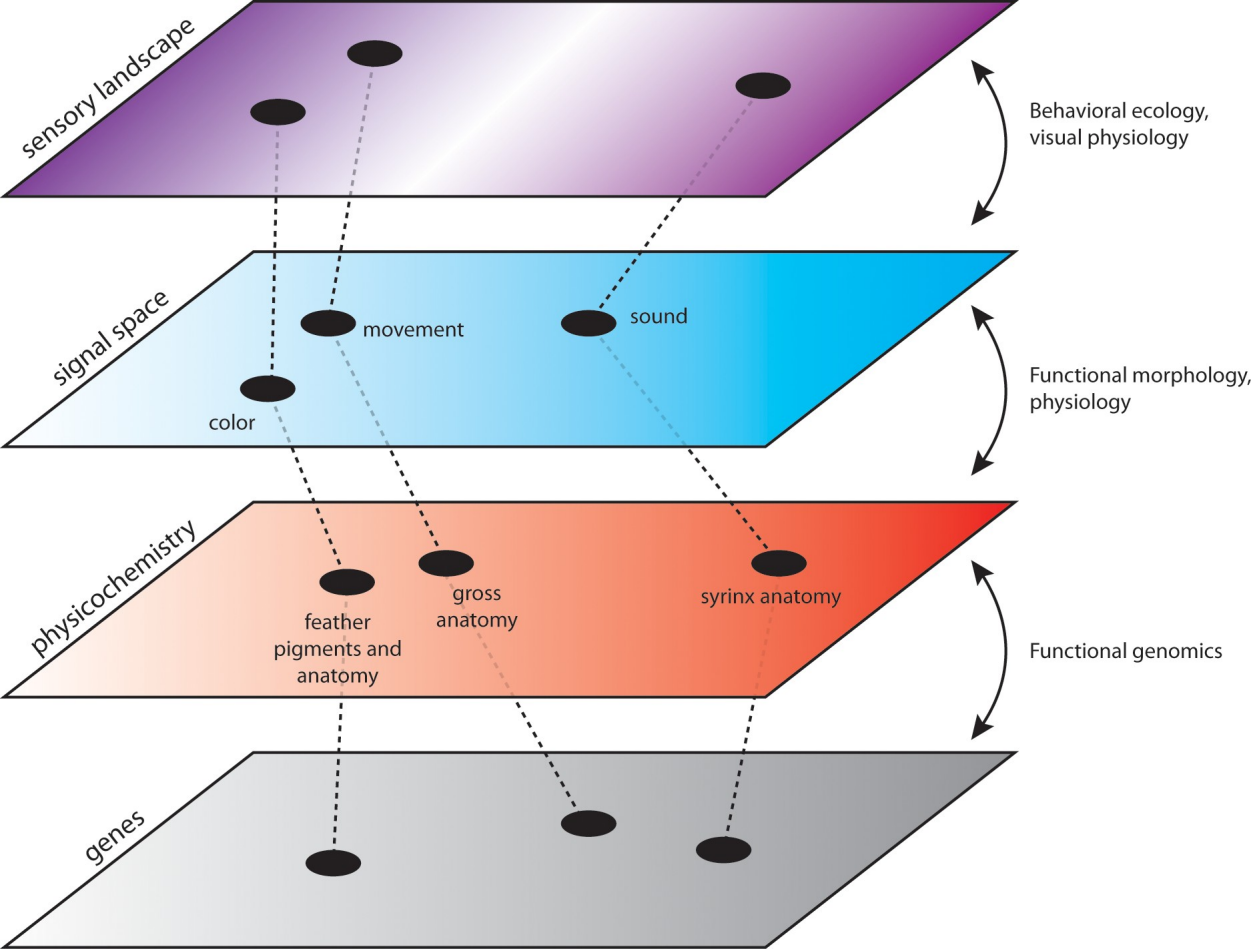
Signal complexity and evolution. An integrative framework for studying the causes and consequences of signal diversity. *Image credit*: *Chad M*. *Eliason*.

A new paper in this issue of *PLOS Biology* [16] uses novel analytical approaches to quantify diversity and richness for distinct aspects of courtship signals (behavioral, acoustic, colorimetric) and test hypotheses for how signal complexity itself evolves, both among species and among signal components (see Fig 1). The diverse birds-of-paradise (BOPs) are an excellent focal group for this work because they provide a classic example of how phenotypic and behavioral diversity is shaped by sexual selection.

A challenge with variable datasets (like BOP signals) is comparing different signals among species. For example, how can we compare a whistle in one species to a chirp in another species? Ligon and colleagues [16] tackle this challenge by tabulating all aspects of signals (for example, individual color patches or movements) across 40 BOP species. Next, they use techniques borrowed from the field of information theory (for example, color pixel clustering) to calculate complexity for distinct axes of signal diversity (acoustic, visual, and behavioral) on a species-by-species basis. For example, a species such as the paradise-crow (*Lycocorax pyrrhopterus*) with a uniformly glossy-black plumage would have a lower visual complexity score than the red bird-of-paradise (*Paradisaea rubra*), a species with an iridescent green throat, red tail, yellow head, and brown back and wings. This composite approach gets around the problem of incomparability in complex signal traits and lets the authors ask the question: How does signal complexity itself change over evolutionary timescales?

Evolutionary tradeoffs involving reduced expression of one trait in response to elaboration of another trait are commonplace in nature. Darwin [17] first discussed the possibility of such tradeoffs in the context of signaling in birds. Recent studies have provided mixed support for this prediction. For example, in cardueline finches, brighter species have simpler songs [18], while in Asian barbets, more colorful species have more complex (longer) songs [11]. These differences could reflect real biological differences among different clades or differences in methodology.

By analyzing signal complexity in unprecedented detail, Ligon and colleagues [16] uncover concerted evolutionary increases in color and acoustic diversity, as well as between behavior and acoustic diversity, within BOPs. That is, species with diverse acoustic signals also have diverse color patterns. These positive relationships in complexity among signal types provide evidence of sexual selection acting on an interrelated set of signal traits, which the authors describe as the "courtship phenotype" [16]. This work highlights a potential role for phenotypic integration (that is, concerted evolutionary changes among traits) in explaining the diverse signals we see in BOPs. Further behavioral and comparative work is needed to determine whether this pattern is unique to BOPs and their distinct evolutionary history or if positive correlations in complexity among signal types are more common in birds than previously recognized.

## Future frontiers in signal evolution

### Identifying the proximate causes of signal divergence

Form–function relationships are well studied in ecological traits [19] but less so in ornamental traits [20,21]. Whether signals evolve by natural or sexual selection, evolutionary changes in signals will depend on underlying physicochemical bases for signal production (Fig 1), such as the evolution of new pigment types [22], variation in hormones that affect signals [23], or anatomical changes in structures or transmission environments used for signaling [20]. Further studies on the proximate bases of signal traits will therefore be crucial to our understanding of how complex signals ultimately evolve and help answer longstanding questions about how signal diversity relates to diversity in signal-generating mechanisms.

### Identifying genomic changes underlying signal variation

Exciting discoveries about how genetic mutations translate to brilliant signals, like red feather ornaments in birds, are now being made with next-generation sequencing technology and genome-wide association studies [24]. Comparative genomic approaches can further be used to identify target genes underpinning novel signals [3]. As access to large datasets continues to grow, researchers will face new challenges with identifying shared aspects of complex signals (that is, signal homology) that are comparable across species. Perhaps individual aspects of complex songs or plumages are not comparable in different species, but certain patterns are (for example, black and white bars, or repeated combinations of notes in a song), akin to how a few developmental precursors might give rise to diverse patterns through reaction-diffusion mechanisms in the context of developmental biology (for example, fur patterns in mammals) [25]. Answers to how signal variation reflects regulatory, genic, or even epigenetic changes within the genome will rely on novel approaches for rapid phenotyping along with new analytical tools for linking genomic changes with complex signal variation.

### Studying evolutionary trends in complex signals

Studying the evolution of single traits shared by a group of closely related species is relatively straightforward. Increases in the number of traits necessary to capture variation in complex signals bring new analytical challenges associated with multivariate datasets. For example, as a general rule of thumb, the number of traits should not be greater than the number of species used in a comparative analysis [26]. Recent methods for studying evolutionary trends in multivariate data that use distance-based methods [26] may get around this limitation. However, we are still left with the challenge of identifying comparable subunits shared by species, which is particularly difficult for complex animal signals. Assessing complex signals on a species-by-species basis and deriving composite scores—for example, describing signal complexity [16] or plumage appearance [27]—is a good starting point for asking questions about how complex signals evolve. As comparative methods continue to expand, for example, in allowing evolutionary comparisons with more traits than species, it will be interesting to test the relative power and inferences these differing approaches allow.

### Understanding how signals evolve on sensory landscapes

Recent work has focused on coevolution of signals and sensory systems or how sensory systems might evolve for optimal perception of signals [28]. However, a well-developed framework for the complementary issue of signal evolution on sensory landscapes is lacking. This might be due in part to difficulties with analyzing complex signals in a comparative framework. We are on the cusp of breakthroughs in this area, with new "phylogenetic natural history" approaches that allow researchers to infer adaptive landscapes from trait data [29]. Future research on how perceptual spaces of receivers [1] might act as sensory landscapes upon which signals can evolve [30] will lead to new discoveries in how sensory landscape influence the direction and rates of change in diverse signals in nature. Advances in the study of signal evolution will also depend on increased availability of signal data via online data repositories, which are at present limited compared to those for morphological data (for example, morphobank.org, morphosource.org), as well as development of rigorous, integrative hypotheses for exploring this fertile area of research.
